# Exploring the validity of routine individuated service data for antenatal HIV surveillance in the Western Cape

**DOI:** 10.21203/rs.3.rs-4065819/v1

**Published:** 2024-03-21

**Authors:** Nisha Jacob, Brian Rice, Alexa Heekes, Leigh F. Johnson, Samantha Brinkmann, Tendesayi Kufa, Adrian Puren, Andrew Boulle

**Affiliations:** University of Cape Town; University of Sheffield; University of Cape Town; University of Cape Town; Western Cape Government: Health and Wellness; National Institute for Communicable Diseases; National Institute for Communicable Diseases; University of Cape Town

**Keywords:** HIV surveillance, sentinel surveys, routine data, HIV prevalence

## Abstract

**Background:**

In the Western Cape, South Africa, public-sector individual-level routine data are consolidated from multiple sources through the Provincial Health Data Centre (PHDC). This enables the description of temporal changes in population-wide antenatal HIV seroprevalence. We evaluated the validity of these data compared to aggregated program data and population-wide sentinel antenatal HIV seroprevalence surveys for the Western Cape province.

**Methods:**

We conducted a retrospective cohort analysis of all pregnancies identified in the PHDC from January 2011 to December 2020. Evidence of antenatal and HIV care from electronic platforms were linked using a unique patient identifier. HIV prevalence estimates were triangulated and compared with available survey estimates and aggregated programmatic data from registers as recorded in the District Health Information System. Provincial, district-level and age-group HIV prevalence estimates were compared between data systems using correlation coefficients, absolute differences and trend analysis.

**Results:**

Of the 977800 pregnancies ascertained, PHDC HIV prevalence estimates from 2011–2013 were widely disparate from aggregate and survey data (due to incomplete electronic data), whereas from 2014 onwards, estimates were within the 95% confidence interval of survey estimates, and closely correlated to aggregate data estimates (r = 0.8; p = 0.01), with an average prevalence difference of 0.4%. PHDC data show a slow but steady increase in provincial HIV prevalence from 16.7% in 2015 to 18.6% in 2020. The highest HIV prevalence was in the Cape Metro district (20.3%) Prevalence estimates by age group were comparable between sentinel surveys and PHDC from 2015 onwards, with prevalence estimates stable over time among younger age-groups (15–24 years) but increased among older age-groups (> 34 years).

**Conclusions:**

This study compares sentinel seroprevalence surveys with both register-based aggregate data and consolidated individuated administrative data. We show that in this setting linked individuated data may be reliably used for HIV surveillance and provide more granular estimates with greater efficiency than seroprevalence surveys and register-based aggregate data.

## Introduction

Monitoring the progress of HIV programmes is reliant, in part, on robust surveillance. Whilst global HIV surveillance has evolved since the beginning of the epidemic(1, 2), survey-based approaches remain the mainstay in many settings. Since 1990, the National Antenatal Sentinel HIV Survey has been conducted among pregnant women at selected public health antenatal clinics in all nine provinces of South Africa annually or biennially(3–5). Until 2015, the surveys were limited to women presenting for their first antenatal visit, but since then follow-up antenatal visits have also been included(3, 6, 7). Due to the national antenatal survey being underpowered to provide accurate estimates at district and sub-district level, the Western Cape Provincial Department of Health expanded the national survey to additional sites from 2001 to 2015 to generate more accurate sub-provincial estimates(4).

In 2013, WHO and UNAIDS published *Guiding principles on ethical issues in HIV surveillance* indicating that unlinked anonymous testing should only be used where there is demonstrable inadequacy of program data for surveillance purposes(8). The WHO 2015 *Consolidated guidelines on HIV testing services*, and the WHO 2022 *Consolidated guidelines on person-centred HIV strategic information*, recommend moving towards using routine programmatic data for antenatal HIV surveillance(8–10). To facilitate this transition in surveillance strategy, evaluations of routine data are required. A 2020 assessment of the national prevention-of-mother-to child programme concluded that South Africa was close to achieving the transition to routine data surveillance in relation to HIV testing, but that further evaluation of data completeness and accuracy was required(11, 12). While these studies look specifically at the validity of routine HIV testing data in comparison to laboratory-based HIV testing data from surveys, it should be emphasised that HIV patient management in South Africa is based on routine HIV point-of-care testing results. Reassuringly, available literature shows high antenatal HIV testing coverage in South Africa of up to 98% by 2011(13). Use of routine data for surveillance purposes requires further validation with other surveillance data sources.

In the Western Cape province of South Africa there are two routine programmatic health information systems related to maternal and HIV care. Aggregated provincial data captured from service-based registers are housed in the District Health Information System (DHIS)(14). The HIV testing services register comprises 46 reporting elements (manual and calculated) for key HIV indicators. The Western Cape Provincial Government has additionally developed a Provincial Health Data Centre (PHDC) in which all individual-level routine data captured electronically in the province are consolidated on a single platform, leveraging the patient folder number as the unique patient identifier(14, 15). This allows linkage of various information systems, including laboratory, pharmacy, and patient administration data, providing a rich source of individuated health information, in the absence of routine electronic patient health records. Within this environment, disease-specific patient cascades (virtual cohorts), such as the HIV care cascade and maternity cascade, may be developed using specific markers of care at different points(14).

Several studies in low- and middle-income countries have compared antenatal survey estimates with routine HIV data(8, 16–21). A 2013 study in the Western Cape showed comparable estimates between antenatal survey data and aggregated antenatal HIV data, but also reported disparities at the sub-district level(4). To date there have been no studies in the South African context comparing antenatal survey HIV estimates with individuated routine data estimates. We sought to evaluate the validity of routine HIV prevalence estimates in pregnant women in the Western Cape Province of South Africa as compared to sentinel surveillance.

## Methods

The study was set in the Western Cape province of South Africa, which is comprised of one metropolitan district, Cape Metro and five less urbanised districts (Overberg, Garden Route, Central Karoo, West Coast and Cape Winelands). We compared cross-sectional HIV antenatal survey estimates to those derived from aggregated program data and individuated program data 2011 to 2020. To evaluate the validity of routine HIV prevalence estimates in pregnant women we analysed four datasets; these are described below.

## National antenatal survey data

National sentinel antenatal survey HIV prevalence estimates for the Western Cape province were obtained from the South African National Department of Health. District-level and age-disaggregated estimates were provided on all pregnant women attending their first antenatal visit in a public health facility in the Western Cape during a 6-week survey period in the years 2011–2015. Post 2015, the national antenatal survey was conducted biennially. In years 2015, 2017 and 2019, the national survey data included pregnant women attending first antenatal visits or follow-up antenatal visits in a public health facility during a 6-week period. However, to promote consistency with earlier surveys, we limit our estimates to first antenatal visit. Blood specimens of survey participants were tested for HIV at a central laboratory using HIV ELISA tests.

## Expanded provincial antenatal survey data

Provincial sentinel antenatal survey data 2011–2015 (subsequently discontinued), incorporating larger, proportionally weighted sample sizes, were obtained from the Western Cape Department of Health. To attain provincial estimates, sub-district data were weighted using the proportional distribution of antenatal first visits in the prior year.

### DHIS (Routine aggregated HIV program data)

Aggregated program data included all women attending their first antenatal visit at primary health care facilities in the Western Cape 2011–2020. All women, regardless of HIV status, are offered a point-of-care HIV test at first antenatal visit. Results of these tests are captured in facility-based registers, initially prevention-of-mother-to-child transmission (PMTCT) register and later primary health care services register. As PMTCT register data were limited from 2011–2013, provincial estimates from 2011 and 2012 were obtained from a study comparing routine aggregated data to sentinel surveys(4). From 2014 to 2020, the proportion of pregnant women at first visit with evidence of prior HIV diagnosis or new diagnosis, derived from the primary health care services register, was used to calculate prevalence estimates. “HIV positive PMTCT initial test” and “Known HIV positive client” elements were combined as the numerator, with total “Antenatal first visit” as the denominator. Routine HIV testing is based on a point-of-care HIV testing algorithm conducted by an HIV counsellor or nurse, where only discordant test results are confirmed by central laboratory-based ELISA testing. Known HIV positive status is captured by an HIV counsellor or nurse based on self-reported HIV and medical records confirming HIV diagnosis.

### PHDC (Routine individuated HIV program data)

A retrospective cohort was derived from the PHDC which included de-identified linked data of all pregnant women attending public health facilities across the province 2011–2020. The cohort was enumerated using the PHDC maternity cascade which links electronic records of all patients with administrative or laboratory evidence indicative of pregnancy. Pregnancies inferred with high confidence were included, as they have at least one high confidence evidence such as a rhesus antibody test (conducted routinely at first antenatal visit), pregnancy test, International Classification of Diseases (ICD) Tenth Revision code indicating pregnancy or pregnancy outcome, maternal discharge summary or repeat moderate confidence evidences such as antenatal visits. Since information specific to the first visit is not routinely captured in all public health facilities, it was not possible to distinguish between first and follow-up antenatal visits, however all pregnancies were only captured once. District and sub-district for pregnancy was determined by the geographic location of the facility of first pregnancy evidence. The pregnancy period was estimated using the pregnancy outcome date and/or any available evidence on gestational age, with year allocated according to the date of first record of pregnancy. Since HIV diagnosis is based on point-of-care testing, these results are not digitised and therefore not available to the PHDC. In the absence of these testing data, administrative, laboratory and pharmacy evidence of HIV diagnosis before or during the estimated pregnancy period was used to determine antenatal HIV status of women in the maternity cohort. The proportion of pregnancies with electronic evidence of HIV diagnosis prior to pregnancy or during pregnancy amongst all pregnant women was used to calculate antenatal HIV prevalence estimates.

#### Analysis

Data were analysed using Microsoft Excel and Stata 17 (Stata Corporation, College Station, Texas, USA). Measures of central tendency and dispersion were used to describe continuous variables, depending on distribution. Categorical variables were described using proportions and 95% confidence intervals, using the normal approximation to the binomial distribution. Descriptive characteristics of the PHDC cohort (2014–2020) were validated with 2014 provincial antenatal survey data. Prevalence estimates from provincial surveys, and aggregated and individuated program data, were calculated for comparison with national survey estimates, serving as the gold standard in this comparative analysis. Data were analysed by year at provincial and district level for comparison.

Provincial estimates from all datasets were further compared with provincial antenatal HIV prevalence estimates from the Thembisa mathematical model(22). This is an integrated demographic and HIV model for South Africa, calibrated to a number of HIV data sources including antenatal HIV prevalence surveys(22). Quantitative comparisons were analysed using correlation coefficients and average percentage differences. Individuated data from 2015 onwards were further disaggregated by district and age for prevalence estimates. Provincial age-group estimates were compared between PHDC and the national antenatal survey from 2015. There were no comparators available for district-level age group estimates as the national antenatal survey is underpowered for this purpose. Data were categorised using age categories routinely used in antenatal survey reporting. The DHIS estimates (2011–2020) and survey estimates (2011–2015) are limited to pregnancies registered for antenatal care, whereas PHDC estimates (2011–2020) include all pregnancies.

#### Ethical considerations

The study was approved by the University of Cape Town Human Research Ethics Committee (HREC 083/2021) and the Western Cape Provincial Health Research Committee. All antenatal HIV sentinel survey and DHIS data were received as aggregates. Data from the PHDC were de-identified before release for the study according to the Western Cape Department of Health Data Access Policy Guidelines.

## Results

From 1 January 2011 to 31 December 2020, 977 800 and 989 568 pregnancies were enumerated by the PHDC and DHIS, respectively. Table 1 presents study population size per year compared with sample sizes of both provincial and national antenatal surveys. As compared to the DHIS, fewer pregnancies were enumerated by PHDC prior to 2015.

**Figure 1:**
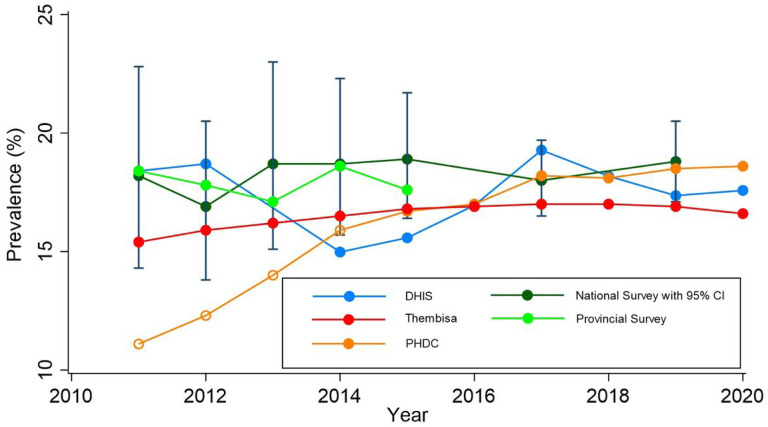
Western Cape Antenatal HIV Prevalence 2011 – 2020 by dataset

Amongst women presenting for their first antenatal visit as recorded in the DHIS aggregated data, the percentage known to be living with HIV increased from 9.5% (95% CI 9.4–9.7%) in 2014 to 15.0% (95% CI 14.8–15.2%) in 2020. Over the same period, the percentage of women accepting an HIV test at first antenatal visit declined from 90.4% (95% CI 90.2–90.6%) to 81.0% (95% CI 80.8–81.2) (Table 1). The characteristics of the women participating in the PHDC cohort (2014–2020) and the 2014 antenatal survey (unweighted) are shown in Table 2. As seen in Table 1 and Fig. 1, PHDC HIV prevalence estimates from 2014 onwards are closely aligned to national, provincial and DHIS data.

The average difference in antenatal HIV prevalence between the PHDC and other datasets are shown in Table 3. A positive correlation was observed between PHDC and DHIS (r = 0.8).

District level prevalence estimates are shown in Supplementary Fig. 1. Prevalence estimates between DHIS and PHDC were closely aligned from 2015 to 2020. PHDC and national antenatal survey district estimates were also closely aligned in 2017 and 2019 in all districts except Overberg, with an average percentage point difference of −4.4 from 2015 to 2020.

**Figure 2:**
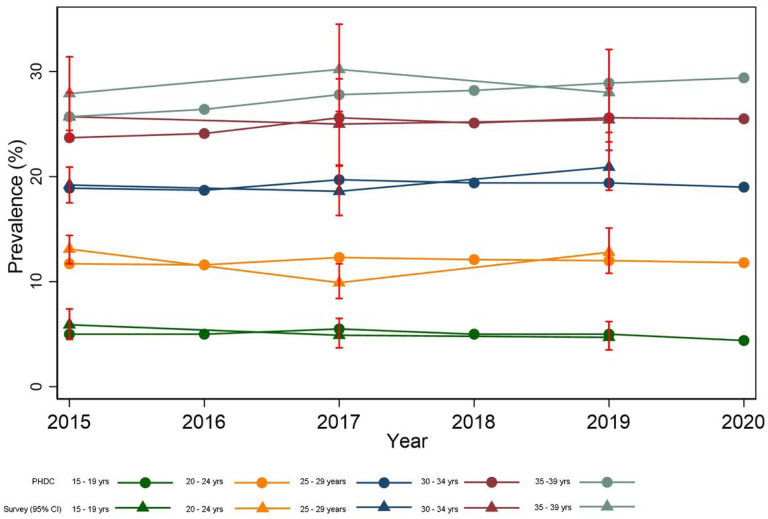
Western Cape Antenatal HIV Prevalence by Age Group: 2015 – 2020 (PHDC and National Antenatal Survey)

HIV prevalence estimates remained stable over time among younger age-groups (aged 15–29 years) but increased among older age-groups (> 34 years). Prevalence estimates between PHDC and national antenatal survey data were closely aligned by age group (Fig. 2).

## Discussion

This is the first study to compare sentinel HIV seroprevalence surveys with both routine aggregated and individuated data. Our results show that from 2015 onwards, the PHDC provides a reliable source of individuated data for accurate and timely antenatal HIV surveillance at provincial, district and age-group levels. These are essential for a responsive health system to plan and evaluate programmes.

From 2015 onwards, the PHDC dataset enumerated more pregnancies than reflected in routine aggregate antenatal first visit registers (DHIS), supporting the completeness of these individuated data. The lower enumeration of pregnancies prior to 2015 is most likely due to limited availability of electronic data for linkage in earlier years, when electronic data systems were less widely established. As more public health facilities began using routine electronic data systems, patients with any contact with public health facilities would have some electronic record enabling inclusion in the PHDC. Given that the routine individuated data represent the entire population utilising public health services, these data are more representative of the population than survey data. It is also likely that linked individuated data are less prone to the biases of routine aggregated data such as duplication and lack of completeness, both numerically and within available records. A single unique identifier across the province prevents duplication of records where patients attend different facilities. Linkage of different electronic systems using a unique identifier also allows more opportunities for ascertainment of pregnancy and HIV status than fixed variables used in aggregate data.

Comparison of descriptive characteristics between PHDC cohort and survey cohort enabled validation of the PHDC cohort prior to estimating HIV prevalence. The median age of women in the PHDC cohort (26.9 years) was similar to that in the antenatal survey population (3, 6), supporting the close alignment in demographic profile of the two cohorts. PHDC, however, had a higher proportion of women with no prior evidence of pregnancy (60%) when compared to recorded gravidity in the survey (approximately 30%)(3, 6). This is likely due to less well-established electronic systems in earlier years for prior pregnancy ascertainment. Furthermore, the PHDC cohort had a higher proportion of patients from the Cape Metro district and lower proportion from the rural districts. This is also likely due to increased PHDC coverage in the Cape Metro, particularly in earlier years as electronic systems were better established in urban areas. The national surveys may further include oversampling of rural districts to generate accurate district-level estimates. We would suggest whole population surveillance negates a need for oversampling.

HIV prevalence estimates in both national and provincial antenatal surveys remained consistent between 2011 and 2015, with provincial surveys providing more precise estimates due to the larger sample size. From 2014 onwards, PHDC estimates are closely aligned to the survey. Given the wiDefconfidence intervals for survey estimates, trends are di cult to infer. DHIS HIV prevalence estimates for 2011 and 2012 were consistent with survey estimates, as shown in a comparative study (4). This study, however, used HIV-service specific denominators from DHIS viz. PMTCT initial HIV test acceptance or refusal. From 2014 onwards, refusal of PMTCT initial test was no longer recorded hence first antenatal visit was used as the denominator, resulting in lower estimates than in earlier years. DHIS data, however, showed an increasing proportion of women over time to be presenting for their first antenatal visit and known to be living with HIV. This increase corresponded with a decreasing proportion of women accepting HIV testing at first antenatal visit. The lower proportion of test acceptance is most likely due to increasing awareness of HIV positive status, in keeping with a recently published modelling study(23). Additionally, some women with known HIV positive status may still retest in antenatal settings, as seen in a recent study in the Western Cape(24). The change in DHIS elements likely represents the changing HIV context with greater awareness of HIV status and wider roll-out of antiretroviral therapy (ART) over time(23). Variability presented in DHIS estimates is likely, in part, due to challenges in maintaining routine aggregate data systems where contextual factors, such as staff turnover, high workload, and social unrest impact data collection and consolidation from registers.

PHDC HIV prevalence estimates 2011 to 2013 were lower than survey estimates, most likely due to incomplete electronic data. From 2014 onwards, estimates were better aligned and more consistent over time. As PHDC estimates do not distinguish between pregnancies with or without antenatal care, prevalence was expected to be slightly higher than that estimated using DHIS and survey data, as these data are limited to pregnancies with antenatal care. Antenatal first visit coverage is however high in the Western Cape at 94% and therefore the contribution of pregnancies without prior antenatal care to HIV prevalence is small in the PHDC estimates(13, 25, 26). Reassuringly, PHDC estimates remained within the 95% confidence intervals of the national survey, and PHDC and DHIS estimates showed a positive correlation and low average difference. It should be noted, however, that average difference may mask the volatility of prevalence trends over time. Low average difference between survey and PHDC estimates suggest the PHDC may be reliably used to estimate antenatal HIV prevalence at provincial level. PHDC estimates over time were also similar to the Thembisa model estimates. Since the Thembisa model includes both private and public sector data, lower estimates than the PHDC were expected (reflecting lower HIV prevalence among private patients)(27).

At a more granular level, from 2015 onwards, estimates from PHDC were closely aligned to both DHIS and survey at district level, with disparities noted more in sparsely populated rural districts like Overberg. These disparities may reflect survey under-sampling in smaller rural districts as well as differences brought about by migrant populations such as workers(28). Age-group HIV prevalence estimates from the PHDC were closely aligned to the national survey from 2015 onwards, showing consistent HIV prevalence in all age groups over a 5-year period, with higher estimates in older age groups. Higher prevalence in older age groups is expected due to a combination of factors, including increased cumulative incidence with age, and use of ART extending life expectancy. Since DHIS cannot provide age-disaggregated estimates, comparisons with PHDC and survey could not be made. Furthermore, in recent years, estimates at sub-district level are only possible with the PHDC data as the national survey is underpowered at sub-district level. This again highlights the advantages of linked individuated data over both survey data and aggregate routine data in providing granular estimates, not limited to predetermined indicators.

### Limitations

A comparative study of this nature is subject to several limitations. Firstly, routine data were validated against sentinel surveillance data, with national surveys serving as the most accurate HIV prevalence estimates or “gold standard”. These survey estimates are however based on smaller sample sizes, over a limited time period, and underpowered for granular estimates which may impact accuracy. PHDC data were less reliable before 2014 due to incomplete electronic data in earlier years. Estimates from each dataset are derived using differing numerators and denominators with differing levels of quality and completeness. Furthermore, both individuated and aggregated routine data are subject to various pitfalls such as capturing errors, administrative errors and consolidation errors which may impact quality and completeness of these data. Pregnancy ascertainment may differ between districts and sub-districts due to differing use of electronic information systems. Since detailed patient characteristics such as socio-economic status and education level are not captured routinely, in-depth comparisons with survey data were not possible. Contextual factors impacting on observed trends in routine data are diverse and require further investigation – these incluDefclerical changes in capturing approaches, widespread impact of the COVID-19 pandemic on service utilisation and staffing, migration patterns etc. Survey and routine data sources evaluated in this study include only patients utilising public health care services, excluding those in the private sector and those without access to public health care. Lastly, while the Western Cape province has established individuated data systems, most other provinces in South Africa are still reliant on aggregated register-based data. The results of this study are therefore not representative of the whole country and highlight the need and potential to strengthen *individuated routine* information systems for improved surveillance.

## Conclusion

Our study demonstrates the validity of routine individuated data for timely and efficient HIV antenatal surveillance, without the additional cost and logistical complexity brought about by regular surveys and with fewer biases compared to routine aggregated data. We highlight the added utility of routine individuated data in providing more granular estimates than sentinel surveillance at district and sub-district level, thereby facilitating more detailed and timely population-level epidemiological trend analysis. While provincial antenatal HIV prevalence trends have increased slowly over time, notable differences in district level trends require further investigation. Strengthening of routine individuated data systems will create an actionable platform to support service delivery and allow richer, more efficient, less costly and more timeous HIV surveillance.

## Figures and Tables

**Table 1:** Antenatal HIV prevalence estimates by dataset

	Pregnancies (N)					HIV testing (DHIS)		HIV prevalence§					
	National Survey	Provincial Survey	DHIS	PHDC	DHIS/PHDC difference	Known HIV positive among all first antenatal visits (%)	Accepted testing among all first antenatal visits (%)	National Survey (%, 95% CI)		Provincial Survey (%, 95% CI)		DHIS (%)	PHDC (%)
2011	4044	9812	97588	46703	−50885			18.2	14.3–22.8	18.4	17.7–19.2	18.4‡	11.1
2012	4010	8711	97144	59826	−37318			16.9	13.8–20.5	17.8	16.7– 18.3	18.7‡	12.3
2013	3793	8125	96993	81761	−15232			18.7	15.1–23.0	17.1	16.4–18.0		14.0
2014	4036	7480	99454	94200	−5254	9.5	89.7	18.7	15.7–22.3	18.6	17.7–19.4	15.0	15.9
2015	7517†	7560	92168	101730	9562	9.8	90.1	18.9	16.4–21.7	17.6	16.8–18.4	15.6	16.7
2016			90034	106256	16222	11.8	90.4					17.0	17.0
2017	3571		95334	115704	20370	15.1	89.2	18.0*	16.5–19.7			19.3	18.2
2018			101044	120547	19503	14.6	84.7					18.2	18.1
2019	3943		110145	123907	13762	14.3	82.6	18.8**	17.1–20.5			17.4	18.5
2020			109664	127166	17502	15.0	81.0					17.6	18.6

† 2015 national survey included additional Western Cape data and was limited to women presenting for first antenatal visit.

‡ 2011 and 2012 DHIS estimates denominator was total number of women refusing or accepting PMTCT initial tests whereas later estimates used the denominator of total antenatal visits

* Prevalence estimate is among first antenatal visit only. 2017 reported prevalence including both first and follow-up attendees is 15.9% (95% CI 14.2 −17.8).

** Prevalence estimate is among first antenatal visits only. 2019 reported prevalence including both first and follow-up attendees is 17.9% (95% CI 16.2 – 19.7).

§ 95% Confidence intervals are not reported for routine data estimates as the whole population is included precluding the need for sampling

**Table 2:** Descriptive characteristics of PHDC Cohort (2014–2020) compared to 2014 Provincial Antenatal Survey

	PHDC Cohort 2014–2020 (n = 789510) Percentage	95% CI	Antenatal survey 2014 (n = 7526) Percentage	95% CI
Electronic evidence of cunent and prior pregnancy*			Gravidity	
**1**	57.9	57.8–58.0	30.4	29.4–31.5
**2**	28.0	27.9–28.1	32.0	30.9–33.0
**3**	10.2	10.2–10.3	21.7	20.8–22.7
**4**	2.9	2.9–3.0	10.1	9.5–10.8
**5 or more**	0.9	0.9–1.0	5.8	5.3–6.3
**Age (median; IQR)**	27.0 (22.6–32.0)		26.4 (22.0–31.0)	
**Age Category**				
**<15**	0.5	0.4–0.5	0.4	0.3–0.6
**15–19**	11.8	11.7–11.9	13.5	12.7–14.2
**20–24**	26.4	26.3–26.5	28.5	27.5–29.5
**25–29**	27.3	27.2–27.4	26.9	25.9–27.9
**30–34**	20.5	20.4–20.6	19.8	18.9–20.8
**35–39**	10.6	10.5–10.6	8.6	8.0–9.3
**>39**	3.0	3.0–3.1	2.2	1.8–2.5
**District** **				
Cape Winelands	13.7	13.7–13.8	15.8	15.0–16.7
Central Karoo	0.97	0.95–1.0	1.9	1.3–1.9
Cape Metro	67.4	67.3–67.5	52.9	51.8–54.1
Garden Route	9.0	8.9–9.0	15.0	14.2–15.8
Overberg	3.6	3.5–3.6	5.5	5.0–6.0
West Coast	4.6	4.5–4.6	9.1	8.5–9.8
No district recorded	0.7	0.7–0.8		

* Gravidity estimates (number of times a woman has been pregnant, including current pregnancy) in the PHDC are not reliable since historic data are incomplete. Electronic evidence of current and prior pregnancy is used as a proxy to provide a full description of the cohort.

** Unweighted

**Table 3:** Comparison of antenatal HIV prevalence between PHDC and other datasets (2014–2020)

Datasets compared	Mean absolute difference	Mean difference	Pearson’s correlation coefficient
PHDC vs DHIS	0.8	0.4	r = 0.8 (p = 0.01)
PHDC vs National Survey	1.4	−1.3	r = − 0.4 (p = 0.480)

## Data Availability

All antenatal HIV survey data, DHIS and Thembisa data were provided in aggregated form. The PHDC and DHIS data used in the study include unconsented, de-identified routine service data housed by the Western Cape Department of Health. Release of these data to a public domain would violate the Data Access Policy of the Western Cape Department of Health. Ethically approved data requests which may be targeting the same or similar data sources may be sent to or discussed with the Western Cape Provincial Department of Health and Wellness: Health.Research@westerncape.gov.za. The survey data are available from the National Department of Health, South Africa. Restrictions apply to the availability of these data, however these data may be requested from the National Department of Health, South Africa at adrianp@nicd.ac.za. Thembisa model data can be accessed from https://www.thembisa.org/.

## Abbreviations

ART: Antiretroviral therapy
DHIS: District Health Information System
PHDC: Provincial Health Data Centre
PMTCT: Prevention–of–mother–to–child transmission
